# Codon usage similarity between viral and some host genes suggests a codon-specific translational regulation

**DOI:** 10.1016/j.heliyon.2020.e03915

**Published:** 2020-05-08

**Authors:** Kunlakanya Jitobaom, Supinya Phakaratsakul, Thanyaporn Sirihongthong, Sasithorn Chotewutmontri, Prapat Suriyaphol, Ornpreya Suptawiwat, Prasert Auewarakul

**Affiliations:** a Department of Microbiology, Faculty of Medicine Siriraj Hospital, Mahidol University, Thailand; b Faculty of Medicine and Public Health, HRH Princess Chulabhorn College of Medical Science, Chulabhorn Royal Academy, Bangkok, Thailand; c Division of Bioinformatics and Data Management for Research, Department of Research and Development, Faculty of Medicine, Siriraj Hospital, Mahidol University, Bangkok, Thailand; d Center of Excellence in Bioinformatics and Clinical Data Management, Faculty of Medicine Siriraj Hospital, Mahidol University, Bangkok, Thailand

**Keywords:** Microbiology, Virology, Viruses, Viral genetics, Genetics, Gene expression, Codon usage pattern, RSCU, RNA viruses, Cell cycle, Translation

## Abstract

The codon usage pattern is a specific characteristic of each species; however, the codon usage of all of the genes in a genome is not uniform. Intriguingly, most viruses have codon usage patterns that are vastly different from the optimal codon usage of their hosts. How viral genes with different codon usage patterns are efficiently expressed during a viral infection is unclear. An analysis of the similarity between viral codon usage and the codon usage of the individual genes of a host genome has never been performed. In this study, we demonstrated that the codon usage of human RNA viruses is similar to that of some human genes, especially those involved in the cell cycle. This finding was substantiated by its concordance with previous reports of an upregulation at the protein level of some of these biological processes. It therefore suggests that some suboptimal viral codon usage patterns may actually be compatible with cellular translational machineries in infected conditions.

## 1 Introduction

The genetic code is degenerate. There are 61 triplet codons coding for 20 amino acids and 3 stop codons [1]. Therefore, each amino acid is encoded by several codons, with the exception of two amino acids (methionine and tryptophan). This codon redundancy results in synonymous codon usage, whereby one amino acid is encoded by 2, 4, or 6 codons [2]. Several previous studies have revealed that synonymous codons are utilized with different frequencies and are not randomly used by different genomes or genes. This non-randomness is referred to as codon usage bias [3, 4]. Each species preferentially uses different synonymous codons [5]. This results in a species-specific codon usage bias.

Similarly, there are several tRNA species that carry the same amino acid. These tRNA species are called isoacceptors [6]. Codons and anti-codons in tRNA do not interact in a one-to-one fashion [7]. Base pairing at the third codon position is wobble; for example, G can pair with both cytosine (C) and uracil (U) [8]. It has been demonstrated that tRNA modification directly affects tRNA and mRNA wobble base pairing [9, 10]. Both the available tRNA isoacceptors and tRNA modification change with the cell cycle, and they can be altered by cellular stresses [11, 12]. For efficient protein translation, the codon usage pattern should correlate with the population of available tRNA isoacceptors [13]. In the cellular stress-response, the alteration could enhance the expression of stress-response genes, with the codon usage patterns compatible with the changed tRNA modifications. Those genes shown to be regulated by this codon-specific manner are called Modification Tunable Transcripts (MoTTs) [12, 14].

Two major models have been proposed to explain the causes of codon usage bias: mutation pressure, and translational selection [15]. As to mutation pressure, it is believed that GC content is the major factor driving codon usage bias [16, 17]. The high mutation rates of some nucleotides or codons result in nucleotide substitution that might contribute to lower frequencies of some nucleotides and codons [15]. Mutation pressure has been suggested to be the most important factor determining the codon usage bias in human RNA viruses [18, 19, 20]. However, there are correlations between the codon usage bias and other factors related to translation efficiency (such as available tRNAs, mRNA secondary structure, translation elongation rate, and the intragenic and intergenic codon bias) that cannot be explained by mutation pressure. This suggests that translational selection also influences codon usage bias [15]. The translational selection acts on codon usage bias to achieve efficient and accurate translation. The use of codons correlates with abundant tRNAs, resulting in a higher translation rate [21, 22, 23]. A correlation between codon usage bias and abundant tRNAs has been found in prokaryotes (such as *E. coli* [24]) and in some eukaryotes (such as *S. cerevisiae* [25], *C. elegans* [22], *Drosophila* [23], and human [26]). However, rare codons are preferred to encode some specific sets of genes or regions of genes, for instance, to enable protein oscillation in different phases of the cell cycle, slow down protein translation across the membrane, and reduce ribosome jamming and mRNA secondary structure at the 5′end of coding sequences [11, 27, 28, 29]. Therefore, the translational selection acting on the optimization of frequent and rare codon utilization is important in appropriate gene translation.

Viral replication is dependent on the cellular machineries of the host cells. Thus, one would intuitively think that the codon usage of a viral genome should match that of its host in order to be efficiently expressed. Surprisingly, however, most viruses have codon usage patterns that are different from the codon usage preference of their hosts [30, 31, 32, 33]. A previous study indicated an alteration in the cellular tRNA level after the infection of human immunodeficiency virus type 1 (HIV-1) [34]. In contrast, the cellular tRNA level was found to be unchanged following vaccinia and influenza A virus (IAV) infection, whereas an alteration in the polysome-associated tRNA population was observed, particularly the population of polysome tRNA isoacceptors correlated with viral codon usage [35]. These findings suggest that the codon usage pattern and the regulation of translational machineries may influence gene expression in some viruses.

In this study, we investigated the relationship between the codon usage bias of human genes and human RNA viruses. It is generally believed that the codon usage bias of viruses differs from that of human genes; however, various human genes possess various codon usage patterns [19, 26]. In addition, intragenic codon biases had previously been reported in humans and mice [36]. A comparison of the codon usage at the genome level is therefore too generalized; a more precise comparison at the single-gene level may provide a better insight into the viral codon usage bias.

## 2 Results

### 2.1 Principal component analysis of the relative synonymous codon usage (PCA of RSCU)

**Figure 1:**
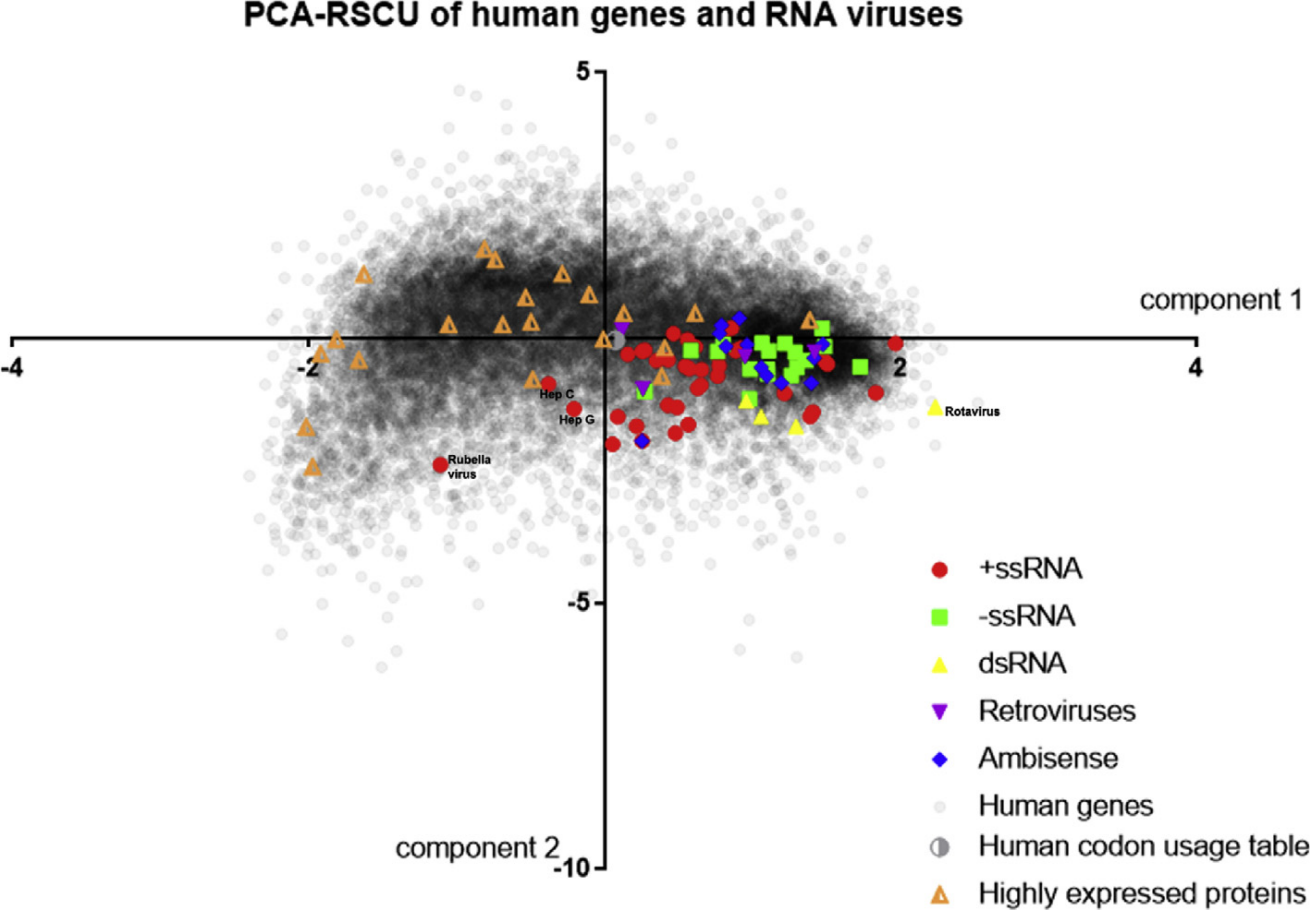
The PCA of the RSCU. The RSCU of human genes and RNA viruses were subjected to PCA. Then, the simplified RSCU values of human genes and RNA viruses were plotted on the graph as the coordinates of component 1 (*x*) and 2 (*y*). The color keys indicate the groups of RNA viruses. Human genes are represented using transparent black dots.

A total of 20,190 major transcript variants of human protein coding sequences were recruited from the GENCODE database (version 26). The protein coding sequences of 77 human RNA viruses were downloaded from the NCBI database. The open reading frames (ORFs) of the protein coding sequences were rechecked by ORFfinder (NCBI) before performing the RSCU calculation. The RSCU is a simple parameter that represents the codon usage bias of synonymous codons in a coding sequence. In our analysis, the RSCU was calculated from the protein coding sequences of the human and RNA viruses. The RSCU of each gene consists of 59 values corresponding to 59 synonymous codons; thus, the PCA was performed to simplify the data to a smaller number of principal factors as a summary feature of the codon usage pattern of each gene. The PCA successfully reduced the 59 values of each RSCU into two significant components. The RSCUs of the human genes and RNA viruses were represented by the coordinates of principal component 1 (PC1, *x*-axis) and principal component 2 (PC2, *y*-axis) plotted on the PCA of an RSCU graph (Figure 1). The RSCU and PCA of the RSCUs of the human genes and human RNA viruses are shown in Supplementary File 1.

In Figure 1, the PCAs of the RSCUs of the human genes were represented with a transparent black dot; genes with a similar RSCU were located in the same area of the graph. The number of human gene located in each quadrant was counted: upper left, 6,020 genes; lower left, 4,227 genes; upper right, 4,664 genes; and lower right, 5,281 genes. Interestingly, many human genes were located densely in the right quadrants, specifically, between (x = 0.95 to 1.7) and (y = -0.7 to 0.6). Most RNA viruses were also located in the right quadrants. Additionally, negative sense-single strand RNA viruses (-ssRNA), ambisense RNA viruses (Ambi), and HIV-1 viruses were located in the area of the right quadrants where the human genes were densely located. However, a great variation was observed in some groups of RNA viruses, especially positive-sense single strand RNA viruses (+ssRNA), doubled strand RNA viruses (dsRNA), and retroviruses (Retro).

**Figure 2:**
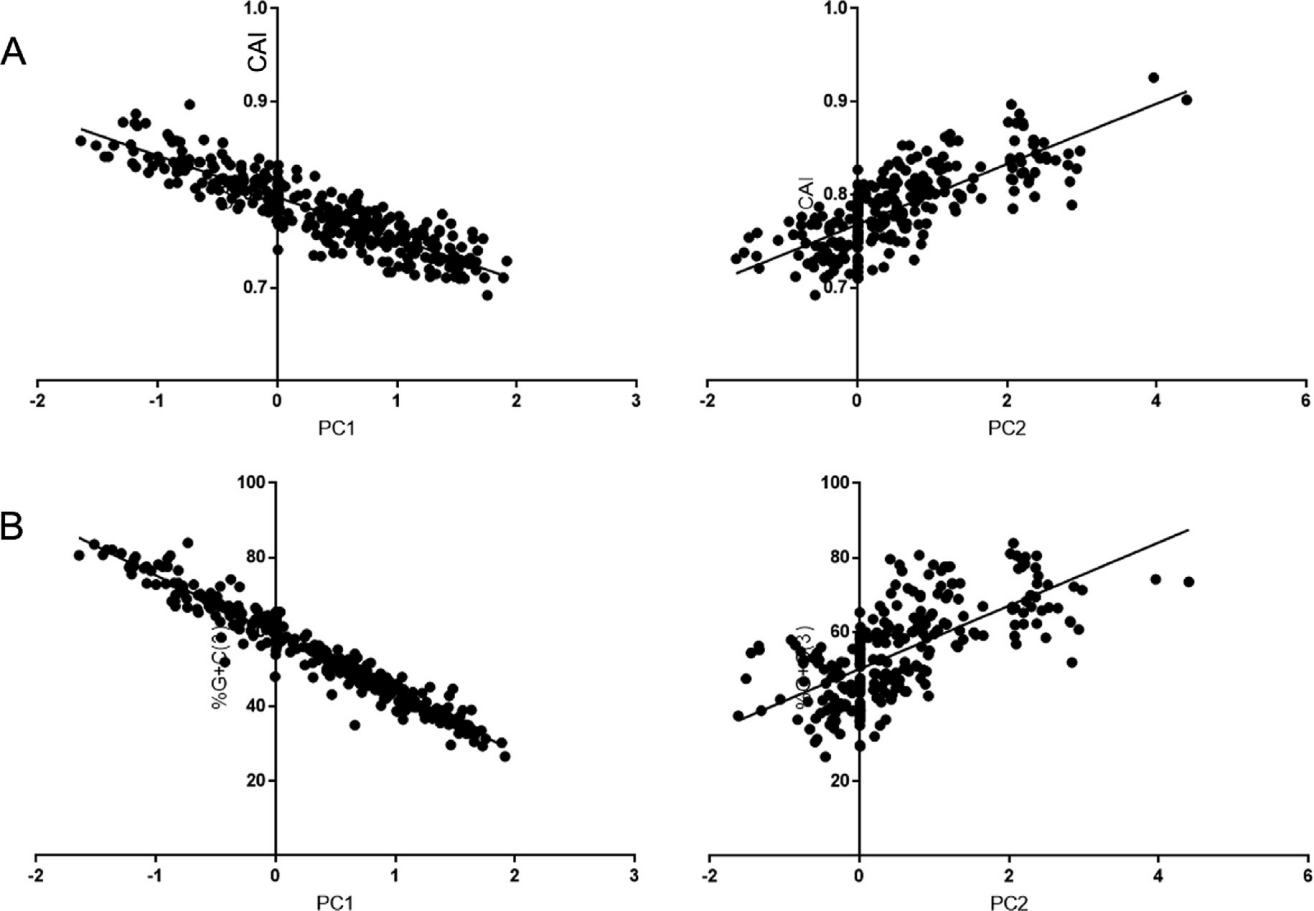
The correlation between principal components (PC1, PC2), codon adaptive index (CAI), and percentage of GC content at the third position of codon (%G+C(3)) were determined using simple linear regression and Pearson correlation coefficient. (A) CAI was plotted with either PC1 (R^2^ = 0.7958, PCC = -0.8921, *p* < 0.001) or PC2 (R^2^ 0.5824, PCC = 0.7631, *p* < 0.001). (B) %G+C(3) was plotted with either PC1 (R^2^ = 0.9459, PCC = -0.9726, *p* < 0.001) or PC2 (R^2^ = 0.4373, PCC = 0.6613, *p* < 0.001).

The relationship between the PCA of the RSCU analysis and the codon adaptive index (CAI) was investigated. CAI is a common parameter used to assess the codon usage bias of a gene. It is calculated from the frequency of the overall codons in a given protein coding sequence with respect to a reference set of genes [37]. In our analysis, the human codon usage table, which is the average codon frequency of a human genome, was used as the reference set. From the results (Figure 1), the PCA of the RSCU of the human codon usage table was plotted near the *x* and *y* intercepts, showing the average codon usage pattern of all human genes. A number of human genes in the PCA of the RSCU graph were selected and subjected to the CAI calculation. The graphs of PC1 and CAI, of PC2 and CAI, were plotted; the linear regression and Pearson correlation coefficient (PCC) were subsequently analyzed. From Figure 2A, it was found that the CAI of genes gradually decreased with an increase in PC1 (R^2^ = 0.7958, PCC = -0.8921, *p*-value <0.0001), while a positive correlation was observed between CAI and PC2 (R^2^ = 0.5824, PCC = 0.7631, *p*-value < 0.0001). The percentages of the GC content at the third position of the codon (%G+C(3)) of the human genes were also determined. In a similar way to CAI (Figure 2B), %G+C(3) gradually decreased with an increase in PC1 (R^2^ = 0.9459, PCC = -0.9726, *p*-value < 0.0001). A weak correlation between PC2 and %G+C(3) was observed (R^2^ = 0.4373, PCC = 0.6613, *p*-value < 0.0001). Thus, the PCA of the RSCU analysis could be used to characterize the heterogeneity of the codon usage bias in the human genome, in which genes in the left-upper quadrant contain more optimal codon usage for high expression, whereas those in the right quadrants near or below the *x*-axis have less optimal codon usage.

The PCAs of RSCUs of the human genes that coded for highly expressed proteins were plotted on a graph. The highly expressed proteins of humans had been previously identified using the proteomic approach (Figure 1; see the gene list in Supplementary Table 1). [38]. From Figure 1, most of the highly expressed proteins were located in the left quadrants. This is in agreement with our analysis showing the relationship between PCA and CAI, and it supports the validity of using PCA to predict CAI.

### 2.2 Human genes with codon usage bias similar to RNA viruses

From the PCA of the RSCU graph (Figure 1), it was demonstrated that most of the human RNA viruses were located in the right quadrants. The degree of difference in the codon usage pattern varied among the groups of viruses. This feature was also observed intragroup. The highest PC1 (*x*) belonged to rotavirus, indicating a high degree of difference in codon usage pattern compared to human genes and other RNA viruses. In particular, there were a number of human genes with RSCUs similar to RNA viruses (Figure 1), especially -ssRNA, Ambi, and HIV viruses, which were located in the right quadrants, where human genes were also located densely. To investigate the kinds of human genes with codon usage patterns similar to RNA viruses and the contributions of those genes in important biological processes, the human genes plotted in the same area with each subgroup of RNA viruses were retrieved. The criteria for selection of the human genes with RSCUs similar to RNA viruses are described in the methods section.

**Figure 3:**
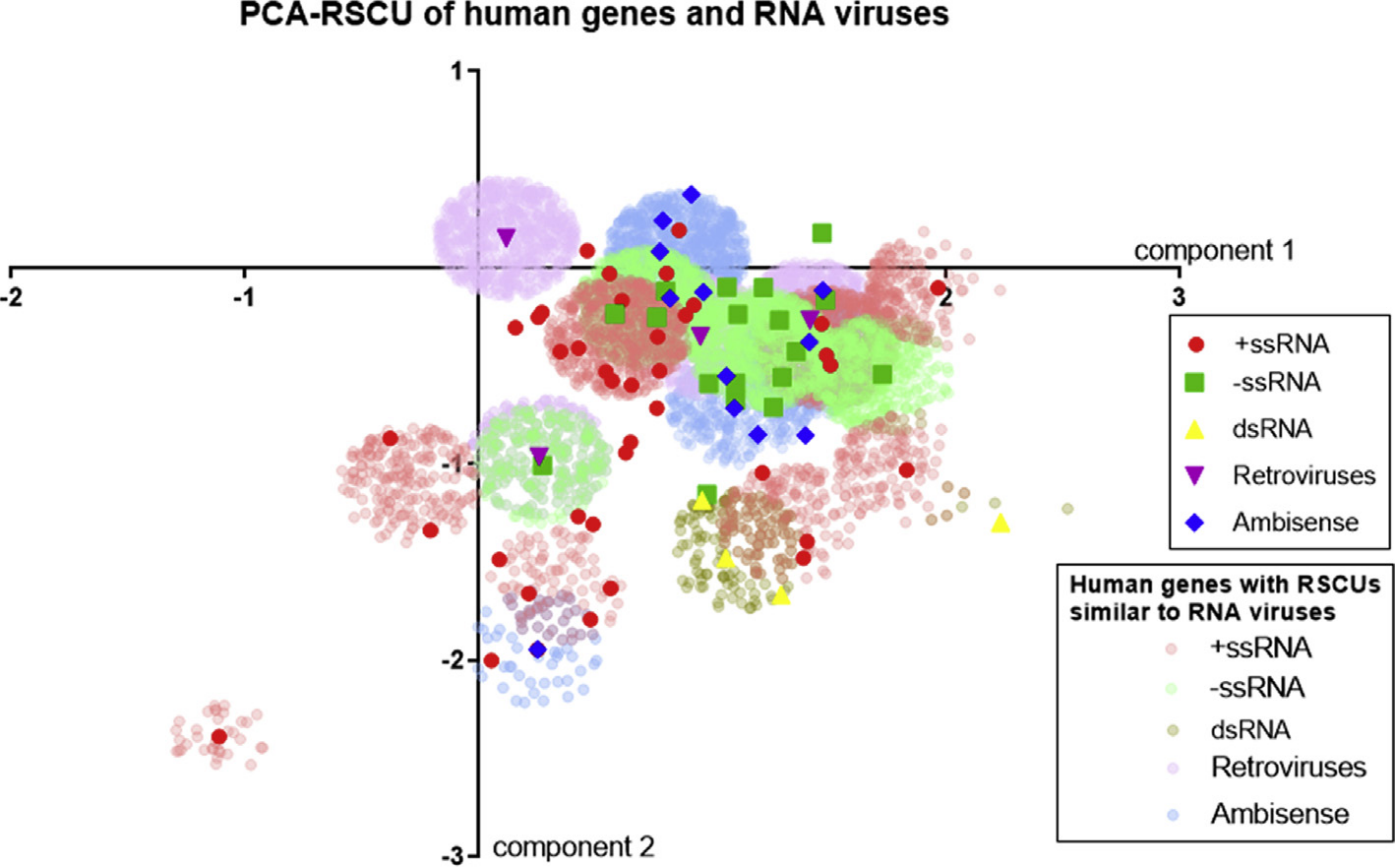
The human genes with RSCUs similar to RNA viruses in each subgroup are represented using transparent colored dots.

**Figure 4:**
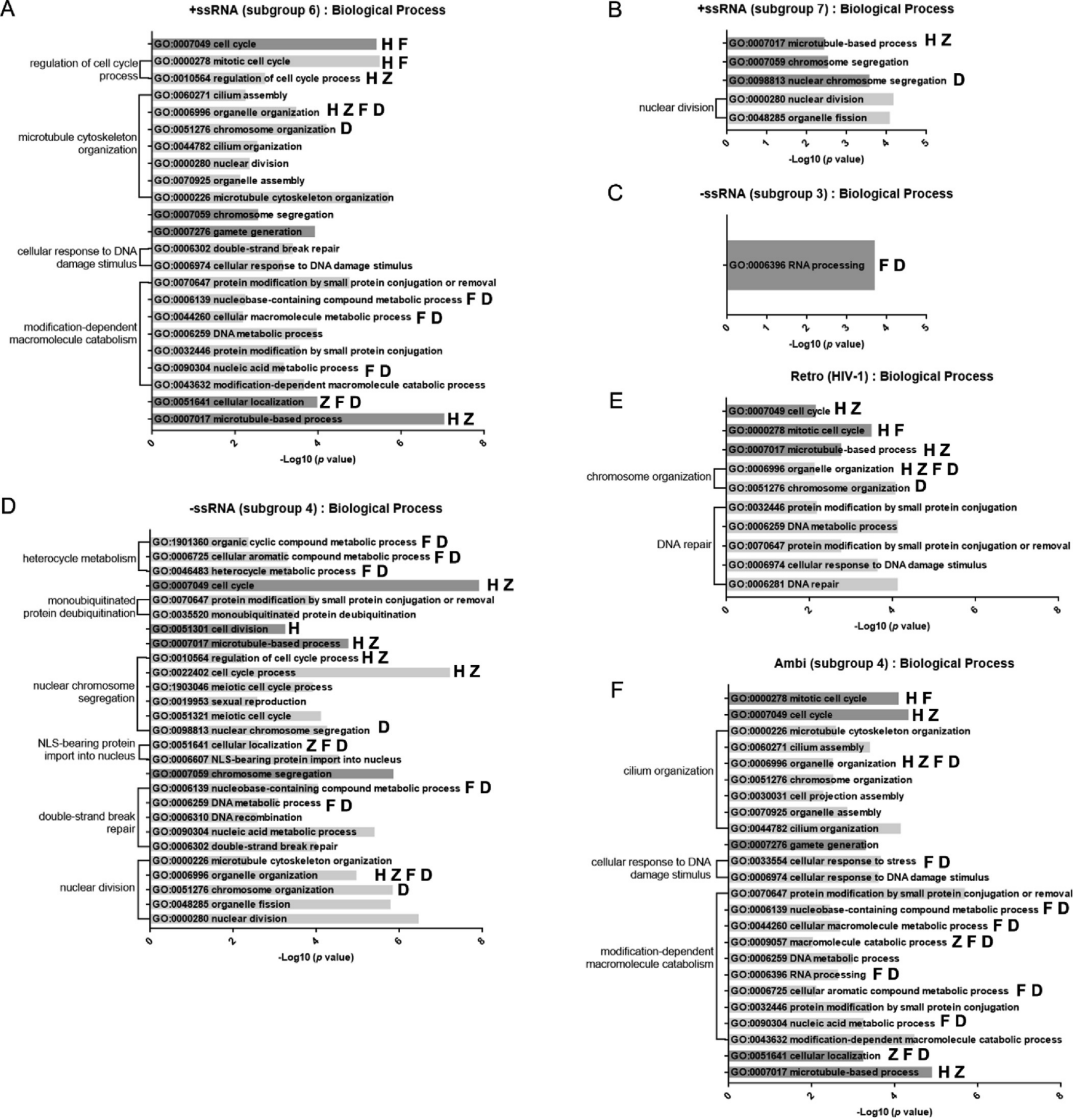
GO terms enrichment in the biological processes from human genes with RSCUs similar to RNA viruses. GO terms with *p* ≤ 0.01 were taken as a significant enrichment and represented with -Log10 (*p-*value). The bold alphabets indicate GO terms that were found to be identical to the enriched GO terms of upregulated protein profiles in viral infections, determined by high-throughput quantitative proteomics; H (HIV-1), Z (Zika virus), F (influenza A virus), and D (dengue virus serotype 2).

Figure 3 represents the selected human genes with RSCUs similar to RNA viruses. These human genes were subjected to gene ontology (GO) enrichment analysis using GO-TermFinder to identify the overrepresented GO terms in biological processes [39]. REVIGO was then used to categorize the redundant GO terms [40]. The number of human genes with RSCUs similar to RNA viruses are listed in Table 1. The results (Figure 4) show the significant GO terms in the biological processes of human genes with RSCUs similar to RNA viruses. Only human genes retrieved from six subgroups of RNA viruses resulted in significant enrichment, namely, +ssRNA (subgroups 6, 7), -ssRNA (subgroups 3, 4), Retro (HIV-1), and Ambi (subgroup 4), where the human genes recruited from +ssRNA (subgroups 6, 7), –ssRNA (subgroup 4), Retro (HIV-1), and Ambi (subgroup 4) were in the same or adjacent area. The human genes with RSCUs similar to +ssRNA (subgroup 6), –ssRNA (subgroup 4), Retro (HIV-1), and Ambi (subgroup 4) shared similar GO terms in biological processes, including the cell cycle, the regulation of the cell cycle process, cell division, microtubule cytoskeletal organization, chromosome segregation, DNA repair, macromolecule catabolism, and cellular localization (Fig. 4A, B, D, E, and F), while human genes with RSCUs similar to +ssRNA (subgroup 3) were related to RNA processing (Figure 4C). The list of enriched GO terms in biological processes is shown in Supplementary File 2.

**Table 1:** Categories of human RNA viruses. RNA viruses were categorized based on the nucleic acid types of their genomes. The viruses in each group were divided into subgroups based on their PCA of RSCU. In subgroups containing more than one virus, the mean virus RSCU (mean vRSCU [a, b]) was calculated. The number of human genes with RSCUs similar to RNA viruses were also represented.

RNA viruses	Subgroups	Members	PCA of RSCU component		No. of human genes with similar RSCU
			1 (*x*)	2 (*y*)	
+ssRNA	**1**	Rubella virus	-1.11152	-2.38693	67
	**2**	Hepatitis G virus	-0.20805	-1.33632	
		Hepatitis C virus	-0.38012	-0.86880	
		**Mean vRSCU**	**-0.29409**	**-1.10256**	192
	**3**	Hepatitis E virus	0.05314	-1.99911	
		Ross river virus	0.08755	-1.48581	
		Chikungunya virus	0.21340	-1.65931	
		Sindbis virus	0.25123	-1.94371	
		Eastern equine encephalitis virus	0.48833	-1.30690	
		Western equine encephalitis virus	0.47744	-1.79137	
		Venezuelan equine encephalitis virus	0.42568	-1.26628	
		O'nyong nyong virus	0.56417	-1.63171	
		**Mean vRSCU**	**0.32012**	**-1.63553**	101
	**4**	Enterovirus 71	0.64889	-0.88714	
		Human coxsackievirus A9	0.54435	-0.52889	
		Human coxsackievirus B4	0.65238	-0.59692	
		Echoviruses	0.57004	-0.57533	
		Polioviruses	0.76484	-0.35123	
		Norwalk virus	0.77184	-0.52468	
		Sapporo virus	0.62754	-0.94239	
		Dengue virus type 1	0.91914	-0.19005	
		Dengue virus type 2	0.85638	0.19119	
		Dengue virus type 3	0.88466	-0.24134	
		Dengue virus type 4	0.80321	-0.02901	
		Japanese encephalitis virus	0.34886	-0.42656	
		Murray Valley encephalitis virus	0.76079	-0.71468	
		St. Louis encephalitis virus	0.61121	-0.16698	
		West Nile virus	0.42713	-0.40840	
		Yellow fever virus	0.55894	-0.03048	
		Zika virus	0.46368	0.08730	
		Kyasanur Forest disease virus	0.26948	-0.22710	
		Omsk hemorrhagic fever virus	0.25430	-0.25169	
		Tick-borne encephalitis virus	0.15574	-0.30465	
		**Mean vRSCU**	**0.59467**	**-0.35595**	659
+ssRNA	**5**	SARS coronavirus	1.38814	-1.47854	
		MERS coronavirus	1.40490	-1.39321	
		Human astrovirus	1.21324	-1.04323	
		**Mean vRSCU**	**1.33543**	**-1.30499**	169
	**6**	Rhinovirus A,B and C	1.46621	-0.28559	
		Human parechovirus	1.48761	-0.44531	
		Enterovirus 68	1.50423	-0.49488	
		**Mean vRSCU**	**1.48602**	**-0.40859**	946
	**7**	Hepatitis A virus	1.96492	-0.10146	188
	**8**	Human coronaviruses	1.83078	-1.03063	129
-ssRNA	**1**	Borna virus	0.27188	-1.00708	233
	**2**	Rabies virus	0.76128	-0.25367	
		Mokola virus	0.79901	-0.11880	
		Measles virus	0.58253	-0.23633	
		**Mean vRSCU**	**0.71427**	**-0.20293**	837
	**3**	Vesicular stomatitis virus	1.10766	-0.23715	
		Influenza A virus H3N2	0.98353	-0.58886	
		Marburg virus	1.29734	-0.55782	
		Ebola viruses	1.09920	-0.67494	
		Influenza B virus	1.28533	-0.26627	
		Human parainfluenza virus type 1	1.09898	-0.58691	
		Human parainfluenza virus type 3	1.48386	-0.16452	
		Human parainfluenza virus type 2	1.25962	-0.71004	
		Mumps virus	0.97594	-1.15319	
		Nipah virus	1.21851	-0.10109	
		Hendra virus	1.06209	-0.09805	
		Respiratory syncytial virus	1.46890	0.17766	
		Metapneumovirus	1.35819	-0.42703	
		**Mean vRSCU**	**1.20763**	**-0.41448**	898
	**4**	Influenza C virus	1.72634	-0.54316	586
dsRNA	**1**	Colorado tick fever virus	1.29334	-1.66493	
		Mammalian orthoreovirus	1.05591	-1.47831	
		Human picobirnavirus	0.95531	-1.18070	
		**Mean vRSCU**	**1.10152**	**-1.44131**	161
	**2**	Rotaviruses	2.23210	-1.29556	7
Retro	**1**	HTLV-1	0.25781	-0.96125	261
	**2**	HTLV-2	0.11613	0.15063	790
	**3**	HIV-1	1.41668	-0.26596	1037
	**4**	HIV-2	0.94712	-0.34939	812
Ambi	**1**	Sin nombre virus	0.25123	-1.94371	70
	**2**	Lymphocytic choriomeningitis virus	0.77488	0.08308	
		Lassa virus	0.78719	0.24056	
		Machupo virus	0.90932	0.37322	
		Rift valley fever virus	0.81849	-0.15444	
		Junin virus	0.96106	-0.12456	
		**Mean vRSCU**	**0.85019**	**0.08357**	950
	**3**	Guanarito virus	1.09370	-0.71405	
		La Crosse virus	1.19379	-0.84942	
		Crimean-Congo virus	1.05851	-0.55100	
		**Mean vRSCU**	**1.11533**	**-0.70482**	529
	**4**	Bunyamwera virus	1.39643	-0.85192	
		Hantaan virus	1.47251	-0.11594	
		Seoul virus	1.41416	-0.37791	
		**Mean vRSCU**	**1.42770**	**-0.44859**	522

### 2.3 Codon usage bias of cell cycle regulated genes is similar to that of RNA viruses

From the previous section, we demonstrated that human genes in the GO terms of the cell cycle and the regulation of the cell cycle process adopt codon usage patterns similar to those of +ssRNA (subgroups 6, 7), –ssRNA (subgroup 4), retrovirus (HIV-1), and ambisense (subgroup 4) viruses. To confirm that the codon usage patterns of these RNA viruses are similar to human genes in the cell cycle and the regulation of the cell cycle process, the cell cycle codon score (CCCS) was used to evaluate the similarity of the codon usage pattern between that of viral genes and a set of cell cycle-regulated human genes (top-600 set) [11]. The CCCS of human RNA viruses had been calculated and is detailed in Table 2. A positive CCCS indicates that a gene has a codon usage pattern similar to the top-600 set. The results revealed that most of the +ssRNA viruses had a negative CCCS score. Only some +ssRNA viruses had a positive CCCS score (such as dengue viruses [DENVs], MERS-coronavirus, SARS-coronavirus, human coronaviruses, human enterovirus 68, and the hepatitis A virus), whereas most of the –ssRNA, dsRNA, HIV-1, HIV-2, and ambisense viruses had a positive CCCS score. However, HTLV-1 and HTLV-2 in the retrovirus group gave a negative CCCS.

**Table 2:** Cell-Cycle Codon Score (CCCS) of human RNA viruses.

Groups	Gene isoform or viruses	PCA components		CCCS
		PC1 (*x*)	PC2 (*y*)	
+ssRNA	Rubella virus	-1.11152	-2.38693	-0.041109
	Hepatitis G virus	-0.20805	-1.33632	-0.012674
	Hepatitis C virus	-0.38012	-0.8688	-0.015136
	Hepatitis E virus	0.05314	-1.99911	-0.006142
	Ross river virus	0.08755	-1.48581	-0.011565
	Chikungunya virus	0.2134	-1.65931	-0.008180
	Sindbis virus	0.25123	-1.94371	-0.009176
	Eastern equine encephalitis virus	0.48833	-1.3069	-0.002974
	Western equine encephalitis virus	0.47744	-1.79137	-0.003173
	Venezuelan equine encephalitis virus	0.42568	-1.26628	-0.003554
	O'nyong nyong virus	0.56417	-1.63171	-0.001119
	Enterovirus 71	0.64889	-0.88714	-0.000267
	Human coxsackievirus A9	0.54435	-0.52889	-0.002991
	Human coxsackievirus B4	0.65238	-0.59692	-0.000998
	Echoviruses	0.57004	-0.57533	-0.002082
	Polioviruses	0.76484	-0.35123	0.001193
	Norwalk virus	0.77184	-0.52468	0.001137
	Sapporo virus	0.62754	-0.94239	0.004792
	Dengue virus type 1	0.91914	-0.19005	0.005112
	Dengue virus type 2	0.85638	0.19119	0.005403
	Dengue virus type 3	0.88466	-0.24134	0.004578
	Dengue virus type 4	0.80321	-0.02901	0.003912
	Japanese encephalitis virus	0.34886	-0.42656	-0.003955
	Murray Valley encephalitis virus	0.76079	-0.71468	0.002322
	St.Louis encephalitis virus	0.61121	-0.16698	0.000160
	West Nile virus	0.42713	-0.4084	-0.003391
	Yellow fever virus	0.55894	-0.03048	-0.000690
	Zika virus	0.46368	0.0873	-0.002752
	Kyasanur Forest disease virus	0.26948	-0.2271	-0.006086
	Omsk hemorrhagic fever virus	0.2543	-0.25169	-0.005482
	Tick-borne encephalitis virus	0.15574	-0.30465	-0.007427
	SARS coronavirus	1.38814	-1.47854	0.027135
	MERS coronavirus	1.4049	-1.39321	0.031220
	Human astrovirus	1.21324	-1.04323	0.014968
	Rhinovirus A,B and C	1.46621	-0.28559	0.013209
	Human parechovirus	1.48761	-0.44531	0.013904
	Enterovirus 68	1.50423	-0.49488	0.013644
	Hepatitis A virus	1.96492	-0.10146	0.022827
	Human coronaviruses	1.83078	-1.03063	0.024501
-ssRNA	Borna virus	0.27188	-1.00708	-0.002858
	Rabies virus	0.76128	-0.25367	0.000205
	Mokola virus	0.79901	-0.1188	0.001436
	Measles virus	0.58253	-0.23633	0.000810
	Vesicular stomatitis virus	1.10766	-0.23715	0.006431
	Influenza A virus H3N2	0.98353	-0.58886	0.008199
	Marburg virus	1.29734	-0.55782	0.000675
	Ebola viruses	1.0992	-0.67494	0.008390
	Influenza B virus	1.28533	-0.26627	0.014146
	Human parainfluenza virus type 1	1.09898	-0.58691	0.015018
	Human parainfluenza virus type 3	1.48386	-0.16452	0.020038
	Human parainfluenza virus type 2	1.25962	-0.71004	0.016267
	Mumps virus	0.97594	-1.15319	0.009273
	Nipah virus	1.21851	-0.10109	0.011326
	Hendra virus	1.06209	-0.09805	0.009457
	Respiratory syncytial virus	1.4689	0.17766	0.020368
	Metapneumovirus	1.35819	-0.42703	0.016291
	Influenza C virus	1.72634	-0.54316	0.021730
dsRNA	Colorado tick fever virus	1.29334	-1.66493	-0.005517
	Mammalian orthoreovirus	1.05591	-1.47831	0.006214
	Human picobirnavirus	0.95531	-1.1807	0.003846
	Rotaviruses	2.2321	-1.29556	0.028856
Retro	HTLV-1	0.25781	-0.96125	-0.006770
	HTLV-2	0.11613	0.15063	-0.007187
	HIV-1	1.41668	-0.26596	0.008663
	HIV-2	0.94712	-0.34939	0.007142
Ambi	Sin nombre virus	0.25123	-1.94371	0.017737
	Lymphocytic choriomeningitis virus	0.77488	0.08308	0.006150
	Lassa virus	0.78719	0.24056	0.007772
	Machupo virus	0.90932	0.37322	0.008089
	Rift valley fever virus	0.81849	-0.15444	0.003661
	Junin virus	0.96106	-0.12456	0.009001
	Guanarito virus	1.0937	-0.71405	0.009082
	La Crosse virus	1.19379	-0.84942	0.007769
	Crimean-Congo virus	1.05851	-0.551	0.006304
	Bunyamwera virus	1.39643	-0.85192	0.018548
	Hantaan virus	1.47251	-0.11594	0.015166
	Seoul virus	1.41416	-0.37791	0.015762

**Figure 5:**
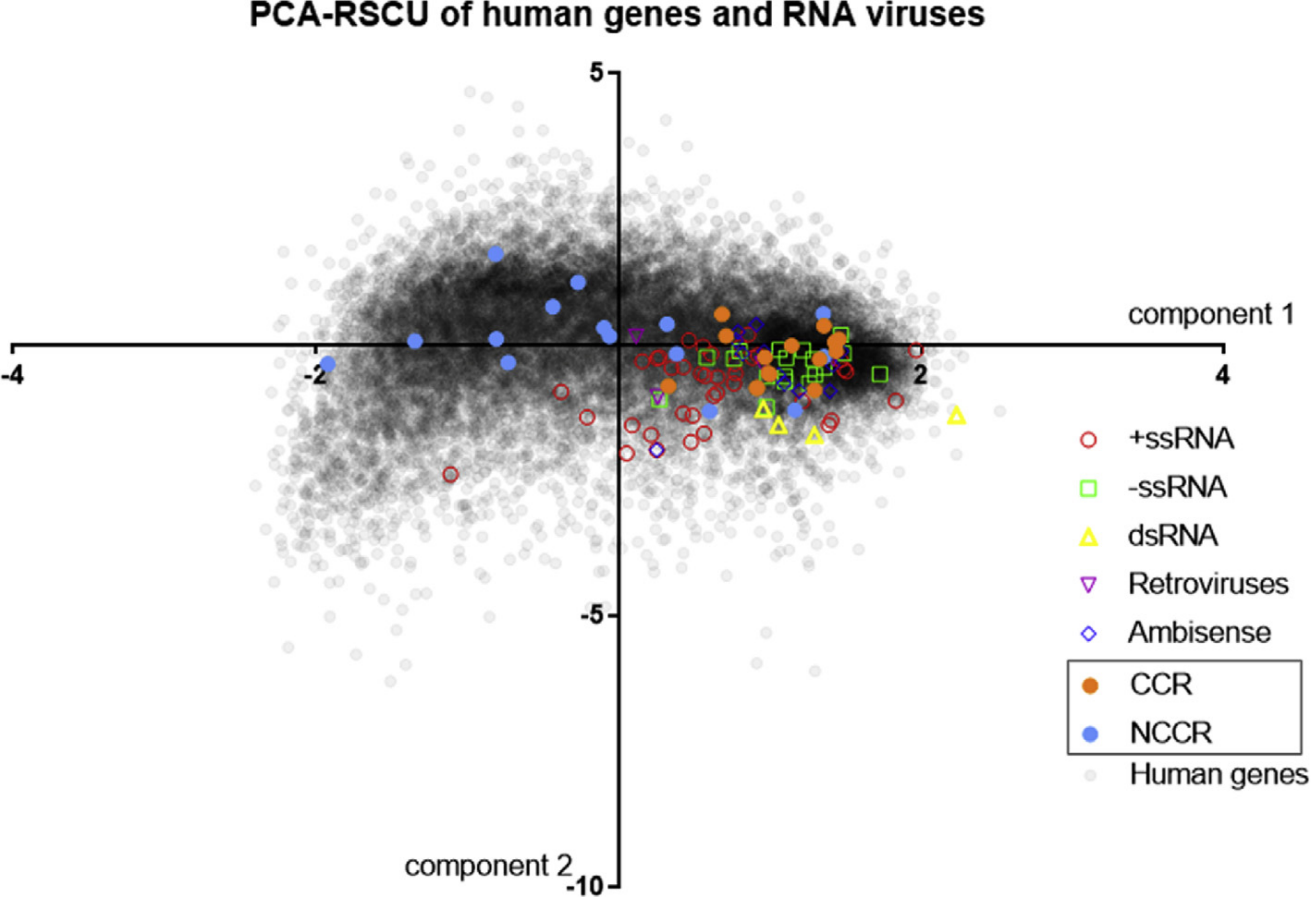
The graph shows the RSCUs of human cell cycle regulated (CCR) genes and non-cell cycle regulated (NCCR) genes, compared with the RSCUs of RNA viruses.

A previous study by Frenkel-Morgenstern *et al.* also demonstrated that the codon usage pattern of the cell cycle regulated genes (CCRs) influence the cell cycle-dependent protein expression [11]. Thus, the similarity of the codon usage pattern of the CCR genes and RNA viruses was investigated. CCR genes cycling at the protein level and non-cycle regulated genes (NCCRs) that were found not to cycle at the protein level were selected from previous studies (Table 3). The RSCUs of the CCR and NCCR genes were plotted on a graph and compared with the RSCUs of RNA viruses (Figure 5). The results showed that the CCR genes were located with -ssRNA, dsRNA, and some +ssRNA viruses, indicating similar codon usage patterns, while the RSCUs of the NCCR genes were distributed all over the graph with no resemblance to RNA viruses. This result corresponds with the CCCS of RNA viruses (Table 2).

**Table 3:** List of CCR and NCCR genes.

	Gene symbols	Descriptions	References
CCR	TRA2B	transformer 2 beta homolog	[70, 71, 72, 73]
	TOP1	topoisomerase	
	E2F5	E2F transcription factor 5	
	H2AFV	H2A histone family member V	
	ANP32E	acidic nuclear phosphoprotein 32 family member E	
	STAG1	stromal antigen 1	
	USP7	ubiquitin specific peptidase 7	
	EZH2	enhancer of zeste 2 polycomb repressive complex 2 subunit	
	RBBP7	RB binding protein 7, chromatin remodeling factor	
	DDX5	DEAD-box helicase 5	
	GTF2F2	general transcription factor IIF subunit 2	
	GARS	glycyl-tRNA synthetase	[11]
	TARS	threonyl-tRNA synthetase	
	EPRS	glutamyl-prolyl-tRNA synthetase	
NCCR	EFHD2	EF-hand domain family member D2	[74]
	ZNF433	zinc finger protein 433	
	STAG3	stromal antigen 3	
	LMNA	lamin A/C	
	E2F4	E2F transcription factor 4	
	HMGA1	high mobility group AT-hook 1	
	YPEL1	yippee like 1	
	SET	SET nuclear proto-oncogene	
	DDX46	DEAD-box helicase 46	
	EZH1	enhancer of zeste 1 polycomb repressive complex 2 subunit	
	HMGA2	high mobility group AT-hook 2	
	SAE1	SUMO1 activating enzyme subunit 1	
	FHAD1	forkhead associated phosphopeptide binding domain 1	
	GAPDH	glyceraldehyde-3-phosphate dehydrogenase	[11]
	WARS	tryptophanyl-tRNA synthetase

### 2.4 Human genes with similar codon usage patterns to RNA virus were upregulated in viral infections

From the previous section, we demonstrated that human genes with codon usage patterns similar to RNA viruses contributed to some important biological processes, such as the cell cycle, the regulation of the cell cycle process, cell division, microtubule cytoskeletal organization, chromosome segregation, DNA repair, macromolecule catabolism, cellular localization, and RNA processing (Figure 4). When coupled with the fact that some viral infections can manipulate host cellular pathways (especially the translation machineries), this finding suggests that human genes with codon usage patterns similar to RNA viruses may be upregulated during viral infection [41].

To substantiate this hypothesis, sets of proteomics data of RNA virus infection were reanalyzed. The lists of upregulated protein profiles upon HIV-1 [42], IAV [43], Zika virus (ZIKV) [44], and dengue virus serotype 2 (DENV-2) [45] infections were obtained from previous studies. The lists of upregulated proteins upon viral infection were submitted to GO-TermFinder (Supplementary File 3), and the enriched GO terms were compared to the enriched GO terms of human genes with codon usage patterns similar to RNA viruses from every subgroup. Several enriched GO terms of upregulated protein profiles during viral infections were found to be identical to the GO terms of human genes with codon usage patterns similar to RNA viruses (Figure 4). In the case of HIV-1 and ZIKV, the identical GO terms included the cell cycle, the regulation of the cell cycle process, the mitotic cell cycle, organelle organization, cell division, microtubule-based process, and cellular localization. The identical GO terms of IAV and DENV-2 included macromolecule metabolic processes, nucleic acid metabolic process, chromosome organization, cellular stress response and RNA processing.

## 3 Discussions

Synonymous codons are distributed unequally and in a non-random fashion, which is referred to as codon usage bias [46]. Moreover, there are significant variations of codon usage bias among different species, and even among genes in the same organism [5, 21]. Theoretically, two major factors shape the codon usage bias: mutation pressure and translational selection [18]. Mutation pressure can result in uneven frequencies of nucleotide content, which can in turn influence codon usage bias [15, 47]. As to translational optimization, the frequent codons are usually found correlated with the population of tRNA isoacceptors [13, 15]. Thus, the frequent or optimal codons would result in more rapid protein translation due to the greater availability of tRNAs corresponding to the frequently used codons [48].

Although the replication of viruses relies on the host cell machinery, several viruses possess different a codon usage pattern to the codon usage preferences of their host [31]. For instance, the HIV-1 genome has been found to be A-rich [49]. The G-to-A hypermutation in the HIV-1 genome has been attributed to viral reverse transcriptase (RT), which lacks 3′ to 5′ exonuclease proofreading activity, leading to the misincorporation of nucleotides [50, 51]. In addition, the function of host enzymes of the APOBEC3 (A3) family has been found to partially contribute to a G-to-A mutation [52, 53]. Furthermore, a difference in codon usage has been observed among individual genes of HIV-1 [51]. The HIV-1 gag gene, encoded for structural protein, adopts a great difference in codon usage pattern compared to human host cells. In contrast, the HIV-1 genes involved in the regulation of the replication cycle, tat and rev genes, have been demonstrated to be more similar to human codon usage bias [54].

In this analysis, the PCA of RSCU represented codon usage patterns of human genes and RNA viruses as a coordinate of PC1 (*x*) and PC2 (*y*) on a graph. We demonstrated that the PCA of RSCU analysis is compatible with a well-established index, CAI. This suggested that the PCA of RSCU could be used in assessing codon usage bias and comparing the difference in codon usage pattern. In particular, the PCA of RSCU allowed a comparison to be made of individual genes in the whole genome scale. Human genes possess various, different codon usage patterns, as observed in each quadrant of a graph. As mentioned earlier, there are a number of human genes located densely in the right quadrants; these genes adopt a non-optimal codon usage pattern similar to CCR genes. Although most of the RNA viruses have a non-optimal codon usage pattern, a great variation in codon usage patterns was observed among the groups of +ssRNA, dsRNA, and retroviruses. The greatest difference in the codon usage patterns belonged to rotavirus, as seen in the graph. By comparison, the rubella virus exhibited a more similar codon usage pattern to humans. These results correspond with those of another study which demonstrated that the codon usage patterns of +ssRNA viruses are closer to human than other RNA viruses, and that the lowest CAI belongs to dsRNA viruses. In more detail, rubella virus (+ssRNA) had the highest CAI at 0.773, and rotavirus had the lowest CAI at 0.683 [55].

A number of human genes with codon usage patterns similar to that of RNA viruses were found by the present study (Figure 3). The human genes with RSCUs similar to RNA viruses were retrieved and subjected to gene ontology enrichment analysis. Interestingly, it was found that only human genes similar to groups of viruses in the right quadrant resulted in significant enrichment, namely, the cell cycle, the cell cycle regulation process, cell division, microtubule cytoskeletal organization, chromosome segregation, DNA repair, macromolecule catabolism, and cellular localization.

The number of human genes with RSCUs similar to RNA viruses retrieved from each subgroup varied from only seven to a thousand genes (Table 1). However, the number of retrieved genes did not affect the significance level or the number of enriched GO terms in Figure 4; for instance, 898 genes for the –ssRNA subgroup 3 resulted in only 1 enriched GO term, whereas 188 genes for the +ssRNA subgroup 7 resulted in 5 GO terms. This suggests that the enriched GO terms did not result from a bias from the different numbers of retrieved genes among the virus groups. It is also possible that some virus groups with a limited number of retrieved genes might not have had sufficient statistical power to enable the detection of enriched GO terms. Among the retrieved human genes, only the genes that adopted a non-optimal codon usage pattern retrieved from the area where they were located densely in the right quadrants gave significant overrepresented GO terms. This suggested that the difference in codon usage bias in human genes might have specific functions. The contribution of non-optimal codon usage bias in human genes on the regulation of protein expression has been investigated in previous research [11, 56, 57]. One study on CCR genes revealed that the non-optimal codon usage pattern generates the oscillation in protein expression during cell cycle progression [11]. We demonstrated that the RSCUs of RNA viruses were similar to the RSCUs of CCR genes, using both the CCCS calculation and the PCA of RSCU. The results showed that –ssRNA, dsRNA, HIV-1, HIV-2, ambisense viruses, and a few viruses in the +ssRNA group exhibited non-optimal codon usage similar to that of the CCR genes. This suggests that despite having a non-optimal codon usage bias, viral genes might be efficiently expressed during the specific phase of the cell cycle correlated with the available tRNA population in that period.

The tRNA population is tissue specific and varies with cellular conditions [58]. Alteration of the tRNA population depends on the level of aminoacyl tRNA synthetase and cellular ATP concentration [59]. In yeast cells, oscillation of the aminoacyl tRNA synthetase and ATP during the cell cycle has been found to result in an increase in tRNA levels in the G2/M phase, but a low tRNA level was observed toward the end of the G1 phase [11]. Therefore, with a low-charged tRNA concentration, the genes expressed during G1 prefer optimal codon usage bias, whereas the genes with non-optimal codon usage bias are highly expressed in the other phases of the cell cycle with a high-charged tRNA concentration [11]. The availability of charged tRNAs during the cell cycle may regulate protein translation in a codon usage-specific manner. Several studies have revealed viral subversion of the cell cycle by arresting via various mechanisms to generate the resources and favorable environment for viral replication and viral protein production [60]. Cell cycle arrest has been observed in both DNA and RNA virus infections. In some RNA viruses, cell cycle arrest at a specific phase may lead to an increase in viral protein translation [61]. During the G2/M phase, the expression of many proteins has been found to fluctuate by arresting at G2/M, viruses may use this mechanism to regulate protein expression [62]. Another study found that HIV-1 was more transcriptionally active during the G2 phase, and that arresting of the cell cycle may help limit the host immune response [63]. Moreover, an HIV-1 infection also causes an alteration at the cellular tRNA level [34]. As to avian coronavirus infections, G2/M arrest has been found with an increased viral protein expression [64]. Furthermore, rotavirus infection arrests the cell cycle in the S/G2 phase, favoring viral protein expression [61], while influenza A virus infection arrests the cell cycle in the G0/G1 phase, resulting in increased viral protein expression [65, 66]. These findings suggest that viruses may manipulate the cell cycle and cellular translation machinery to create available tRNA population favoring the viral codon usage pattern.

Several GO terms of human genes with codon usage patterns similar to RNA viruses have been found by previous studies to be identical to the GO terms of upregulated protein profiles in viral infections. Global proteomic and phosphoproteomic changes in HIV-1 infected CD4+ T cells revealed that HIV-1 affected transcriptional and translational regulation, and targeted RNA or protein degradation in order to modulate biological processes (including signal transduction, cell cycle, metabolic processes, and the immune system) [42]. In addition, a study of ZIKV infected human neurospheres also found an upregulation profile of proteins involving cell cycle arrest. This resulted in an alteration of the cell cycle in order to regulate the transcription and translation of the host cells [44]. IAV infection targeted several cellular pathways to favor its replication, including aminoacyl-tRNA biosynthesis, glycolysis, fatty acid biosynthesis, and spliceosome [43]. Moreover, regulated proteins and phosphoproteins in DENV-infected cells were related to cellular macromolecule biosynthesis, RNA splicing, chromatin modification, and cell stress response, and these regulations help facilitate viral protein expression [45]. This suggests that human genes with codon usage patterns similar to that of viruses may be upregulated at the translational level in a viral infection. It is unclear whether viruses evolved to regulate the translational machinery in order to accommodate the codon usage pattern that had already been shaped by mutational pressure, or whether they adapted their codon usage pattern to match the cellular translational machinery condition in infected cells. These two possibilities are not mutually exclusive. Further studies are required to gain more insight into this new aspect of the virus-host interaction.

## 4 Materials and methods

### 4.1 Protein coding sequences of human genomes

The protein coding sequences of human genomes were downloaded from the GENCODE database (version 26) in FASTA format [67]. The data set provided the nucleotide sequences of the coding transcripts on the reference chromosomes, including multiple transcript variants for each gene. Thus, only the major transcript variants were selected for analysis. The major transcript variant is the longest transcript variant of the gene with a complete ORF. For each gene, one representative as a major transcript variant with the longest sequence length was selected using a custom python script (Supplementary File 4). The ORFs of the protein coding sequences were rechecked by the ORFfinder tool at the website https://www.ncbi.nlm.nih.gov/orffinder (NCBI) before performing further analysis. The transcript variants with an incomplete ORF were excluded and substituted by alternative variants. A list of the selected major transcripts variants is given in Supplementary File 1.

### 4.2 Sequences of human RNA virus genomes

The data set for the sequences of the human RNA virus genome utilized by a previous study was used [55]. A total of 77 RNA viruses that can cause diseases in humans were selected. The protein coding sequences of those RNA viruses were downloaded in FASTA format from the Nucleotide database (RefSeq, NCBI). For each virus species, the protein coding sequences from different isolations of the same virus species were downloaded. The sequences were selected based on their availability in the database (some viral protein coding sequences were only available in a few numbers). Any coding sequence with unidentified nucleotides that could not be translated or with an incomplete ORF was excluded. The selected human RNA viruses were categorized by their family, genus, and genome polarity. They comprised 39 positive-sense single strand RNA (+ssRNA) viruses; 18 negative-sense single strand RNA (–ssRNA) viruses; 4 double strand RNA (dsRNA) viruses; 4 retroviruses (retro); and 12 ambisense (+/-, ambi) viruses. Supplementary File 5 presents a list of the human RNA viruses used in this study, the accession numbers of the sequences, and the number of coding sequences of each virus.

### 4.3 The RSCU analysis

The RSCU is the ratio of the observed frequency of the codon in a gene to the expected frequency of the codon under the condition that all the synonymous codons are equally used. The three stop codons (TAA, TAG, TGA), Met (ATG), and Trp (TGG) were excluded from the analysis. The observed frequency of the codons in the genes was counted. The FASTA sequences were parsed, and the codons of the coding sequences for each transcript variant were counted by a python script and the Biopython library (Python version 3.5.2, with Biopython version 1.66; for the scripts, see Supplementary File 6). Then, the RSCU was calculated as follows:

$$RSCU_{i}=\frac{X_{i}}{\frac{1}{n}\sum_{i=1}^{n}X_{i}}$$

where *n* is the number of synonymous codons (1 ≤ *n* ≤ 6) for the amino acid, and *X*_*i*_ is the number of occurrences of codon *i*. The synonymous codons with RSCU values greater than 1.0 had a positive codon usage bias and were defined as abundant codons, while those with RSCU values less than 1.0 had a negative codon usage bias and were defined as less-abundant codons. In the case of RSCU values that were exactly 1.0, it meant that there was no codon usage bias, and the codons were chosen equally [68]. The RSCU of human RNA viruses were calculated using the CAIcal server, which is available at http://genomes.urv.es/CAIcal [37]. The multiple protein coding sequences from different isolations of the virus in the same species were submitted to the CAIcal server for the RSCU calculation. After that, the average RSCU was calculated to represent the RSCU of each RNA virus species. The RSCUs of the human genes and RNA viruses are provided in Supplementary File 1.

### 4.4 The calculation of CAI

The CAI of a specific gene was calculated using the CAI calculator on the CAIcal server [37]. The reference human codon usage table was obtained from the Codon Usage Database (http://www.kazusa.or.jp/codon/) [69].

### 4.5 PCA of RSCUs

The RSCUs of the protein coding sequences of 20,190 human genes and 77 human RNA viruses were input to the PCA. The PCA was performed using PASW Statistics for Windows, version 18 (SPSS Inc., Chicago, Ill., USA). The Kaiser–Meyer–Olkin Measure of Sampling Adequacy test (KMO–MSA) was also analyzed. The overall KMO–MSA was 0.799, which was greater than a cut off of 0.5, indicating that the sample size was adequate. The principle components were successfully extracted using covariance matrix and Quartimax rotation, which reduced the high dimensions of the dataset to a smaller number of dimensions. The selection of the significant components was based on a scree plot and the proportions of variance. The scree plot was a plot of the component numbers and eigenvalues; only the first two components had eigenvalues greater than 1, and they accounted for 45.5% of the total variance. The RSCU of the gene was represented by the coordinate of PC1 and PC2 (x, y) on the graph. The PCAs of the RSCU graphs were plotted and analyzed using GraphPad Prism 7 (GraphPad Software, Inc., CA).

### 4.6 The CCCS calculation

The CCCS of a gene evaluates the similarity of the codon usage between that of a specific gene and a set of cell cycle regulated human genes. The calculation has been previously described [11]. Briefly, the CCCS is the sum of the codon preference (CP) values of the cell cycle regulated human genes (top-600 set) over all codons in the coding sequence of a gene, normalized by the length of the cDNA. The CCCS of a specific gene was calculated as follows:

$$\text{CCCS}\left(\text{g}\right)=\sum_{\text{codon}\left(\text{gene}\right)}{\text{CP}}^{\text{top}-600}\left(\text{codon}\right)/\text{length}\left(\text{g}\right)$$

where *g* is every codon of a gene, and CP top^−600^(codon) is the CP in the top-600 gene set (see cited reference, Table 1).

### 4.7 GO enrichment

On the PCA of the RSCU graph, the human genes located in the same area of RNA viruses were taken as human genes with RSCUs similar to RNA viruses. To select these human genes, the average RSCU of each group of RNA viruses was initially calculated. However, a great variation of RSCU was observed in each group of RNA virus categorized by the nucleic acid types of their genomes. Hence, the viruses in each group were divided into subgroups by their RSCU before calculation of the average PC1 (*x*) and PC2 (*y*); these RNA-virus subgroups are listed in Table 1. Subsequently, the average RSCU of the RNA viruses in each subgroup (mean vRSCU, coordinate [a, b]) were set as the circle center of a circle with a radius of 0.3 units (Figure 3). The radius was calculated to be minimal, to not exceed the standard deviation of the distance between the circle center and the human genes, and to cover most of the viruses in each subgroup. The human genes located within the circle were taken as the human genes with RSCUs similar to RNA viruses. The distance (r) from the circle center (a, b) was measured as follows:

(a-x) ^2^+ (b-y) ^2^ = r^2^

where *a* and *b* are the coordinates of the mean vRSCU of each subgroup of RNA viruses, while *x* and *y* are the coordinates of the RSCUs of the human genes.

The human genes with an RSCU similar to each group of RNA viruses were analyzed for GO enrichment using Go-TermFinder, and REVIGO was used to categorize the redundant GO terms [39, 40]. The whole genome of *Homo sapiens* was used as a background list. The overrepresented GO terms in the biological process were investigated in both analyses, with *p* ≤ 0.01 taken as a significant enrichment.

### 4.8 Statistical analysis

The simple linear regression analysis and Pearson correlation coefficient (PCC) were determined using GraphPad Prism 7 with *p* < 0.05 was taken as significant.

## Declarations

### Author contribution statement

Kunlakanya Jitobaom: Conceived and designed the experiments; Performed the experiments; Analyzed and interpreted the data; Contributed reagents, materials, analysis tools or data; Wrote the paper.

Supinya Phakaratsakul: Conceived and designed the experiments; Performed the experiments; Analyzed and interpreted the data; Contributed reagents, materials, analysis tools or data.

Thanyaporn Sirihongthong: Conceived and designed the experiments; Performed the experiments; Analyzed and interpreted the data.

Sasithorn Chotewutmontri, Prapat Suriyaphol: Performed the experiments; Analyzed and interpreted the data; Contributed reagents, materials, analysis tools or data.

Ornpreya Suptawiwat: Analyzed and interpreted the data; Contributed reagents, materials, analysis tools or data.

Prasert Auewarakul: Conceived and designed the experiments; Analyzed and interpreted the data.

### Funding statement

This work was supported by Postdoctoral Fellowship, 10.13039/501100004156Mahidol University, Thailand (Grant no. R01612001, 2017), The Royal Golden Jubilee Ph.D. Program, Thailand (Grant No. PHD/0030/2556), Siriraj Graduate Scholarship, 10.13039/501100004156Mahidol University, and Thailand Research Fund (Grant No. IRN60W0002).

### Competing interest statement

The authors declare no conflict of interest.

### Additional information

No additional information is available for this paper.

## Appendix A Supplementary data

Supplementary File 3 Supplementary File 3

Supplementary File 4-longest seq Supplementary File 4-longest seq

Supplementary File 5 Supplementary File 5

Supplementary File 6-codon_count Supplementary File 6-codon_count
