# Supplementary material for: Codon usage similarity between viral and some host genes suggests a codon-specific translational regulation
Source: Heliyon. 2020 May 8;6(5):e03915. doi: 10.1016/j.heliyon.2020.e03915 (PMC7205639; doi:10.1016/j.heliyon.2020.e03915)
Supplement: Supplementary Table 1 [file mmc7.doc]

**Supplementary Table 1.** Highly express proteins in human cells.

| **Gene symbol** | **Descriptions** | **PC1 *(x)*** | **PC2 *(y)*** | **CAI** |
| --- | --- | --- | --- | --- |
| GAPDH | glyceraldehyde-3-phosphate dehydrogenase | -0.81208 | 1.67703 | 0.847 |
| ENO1 | enolase 1 | -0.4992 | 0.30719 | 0.834 |
| PPIA | peptidylprolyl isomerase A | 0.1283 | 0.46753 | 0.834 |
| EEF1A1 | eukaryotic translation elongation factor 1 alpha 1 | 0.61029 | 0.47318 | 0.767 |
| MYOC | myocilin | -0.28693 | 1.20961 | 0.822 |
| ACTB | actin beta | -1.62822 | 1.20124 | 0.897 |
| TUBB | tubulin beta class I | -0.7396 | 1.47446 | 0.817 |
| TUBB3 | tubulin beta 3 class III | -1.92139 | -0.29761 | 0.879 |
| TUBB1 | tubulin beta 1 class VI | -0.69061 | 0.25488 | 0.817 |
| TUBB2A | tubulin beta 2A class IIa | -1.81522 | -0.02513 | 0.886 |
| TUBB2B | tubulin beta 2B class IIb | -1.6616 | -0.41479 | 0.868 |
| RPS2 | ribosomal protein S2 | -1.05605 | 0.2557 | 0.85 |
| HSP90AB1 | heat shock protein 90 alpha family class B member 1 | -0.01058 | -0.02033 | 0.819 |
| HSPA4 | heat shock protein family A (Hsp70) member 4 | 1.38449 | 0.33774 | 0.753 |
| HINFP | histone H4 transcription factor | -0.53335 | -1.67691 | 0.852 |
| HIST2H2BE | histone cluster 2 H2B family member e | -2.01734 | -2.42409 | 0.878 |
| HIST2H3C | histone cluster 2 H3 family member c | -1.97339 | -2.42409 | 0.814 |
| RPS2 | ribosomal protein S2 | -1.05605 | 0.2557 | 0.85 |
| TP53 | tumor protein p53 | -0.1061 | 0.82001 | 0.821 |
